# The Dentist in His Profession and among the People

**Published:** 1895-12

**Authors:** S. B. Hartman

**Affiliations:** Fort Wayne, Ind.


### The Dentist in His Profession and Among the People.

BY S. B. HARTMAN, FORT WAYNE, IND.

Without doubt, to many persons the subject of the few remarks I am to make on this occasion may seem strange, and the question may be asked in what way it has any relative connection to "How to make a Dental Bridge," or "The Preparation of a Cavity for a Gold Filling," or "The Diagnosis of Dental Disease?" as it may seem far remote from what one would expect an essayist to choose as the basis of a paper at an association assembled for the consideration of questions of interest to persons engaged in the treatment of dental tissue, and the artificial replacement of portions of the same lost through chemical or mechanical action. The boy who applied for admtttance as a dental student to a college, when informed an examination would be required in the branches usually taught in the public schools, in reply asked : " What has geography, arithmetic and algebra to do with filling teeth ? " The answer, to many persons may seem to have been the right idea, and no doubt he could become as proficient in that direction as the ancients of many countries were in the art of carving and the construction of masonry, who knew nothing beyond the mechanical formation of their work, and were satisfied with the little circle in which they moved. But as time advanced the people organized and formed nations, and each individual was a component factor, and nations became in knowledge what its people were disposed to make them. While as individuals they each followed some avocation peculiar to himself, yet this influence was felt by his fellow-man.

Thus has our civilization been advancing until we must reach out to others and come in contact with them, and fulfill our duty as a citizen.

The first thought as to what relation a dentist sustains to his country and profession is his first step as a student of our noble calling, and very often if a boy can make a good kite, or his grandfather was a manufacturer of wheelbarrows, or if the boy could make a good joint with two boards in a carpenter shop, his future life should be that of a dentist. While mechanical skill is of great importance, and I would not in any way depreciate the same, yet if our profession wishes to occupy a position on a high plane, and to stand side by side with those professions whose society it strives to associate with, it must of necessity form its young and coming members, not only of those with an inclination to mechanics, but of those that combine these attainments with a knowledge of scientific and physical laws and a general understanding of those studies that will tend to an education that will admit the student to the society of the learned professions. I rejoice that many of our colleges are making the examination for admission of a high standard, more, I think, after having given the question much thought from all standpoints, that the better the general education of the student, the better will be the dental practitioner, not only as a dentist but as a citizen. In addition to the student possessing an education, I would make morality a consideration of his admittance to our profession, for if in any profession or calling it should be insisted upon, it is that of dentistry.

Coming in contact often with those of a sensitive nature, how important for his welfare and the community in which he lives that his character be above reproach ; hence I may say that character is paramount in the admittance of a student.

After having passed the portals of our profession and advanced from step to step to its various branches, and having met the approbation of his instructors, and been awarded their testimony thereof, our next consideration will be the practitioner.

This is often a position that will make a lasting impression ; his idea of what will advance his practice will cause him to be recommended in the community with a welcome, or, otherwise, his ability not only in the mechanical line, but his position as a citizen will be consdered. It is of great importance that he possesses an education outside of his chosen profession, that he may be admitted, and not only admitted, but sought after, to mingle among the best of society of the place where he may reside.

Not long since, a dentist of great ability and an advocate of a higher standard for his profession, and whose life-work has been in this direction, remarked that it was greatly to be regretted that in our profession we had so few men of attainments beyond that required in the care of teeth. While to some extent this is too true, yet I am happy to say that there are many who are taking advanced standing and are endeavoring to raise dentistry into that sphere it should occupy.

Dental associations have much to accomplish to broaden one in thought ; the mingling of persons of like interest incites a zeal to advance ; local societies have salutary influence on each member, and I would that not only questions pertaining to dentistry be considered, but that history, biography, current events, and similar subjects be the topics for papers and discussions; thus would be presented a field for thought and research.

We all have a peculiar influence on others; we may feel that in our little personality no one is changed, but we are little aware how each day some one is elevated or depressed. Science teaches that no force is lost, but goes on and on. A great writer and a man greatly honored says: "The meeting of persons on the street has an unexplainable influence, and little do we know to what extent it may extend."

Several years past a gentleman said to me: "While I was in Cleveland attending a microscopical association I met a dentist from Detroit who had made the study of microscopy one of his pleasures outside of his professional duties and was a great student along that line." Some time after when visiting in Detroit, it was my privilege to call on this gentleman, and one of my greatest enjoyments of the visit to the city was this call. Little, if anything, was said in reference to dental practice but much of the conversation pertained to the great depths of those things not visible to the naked eye. And it seems to me that great fountains of knowledge had opened to me during the time of my conversation with him.

In our profession we need not be circumscribed, and need not and should not live entirely within the four walls of our office. Not only dental journals should be found in the office of a dentist, but the leading magazines of the day. Far better, a dentist should have one dental journal and two literary magazines, than two dental journals and no literary journal or magazine.

A young man quite proficient in operative dentistry, and in this respect one of the best of his class in college, who stood high in his examination in clinical work, was deficient in chemistry and physiology and failed to graduate with his class. Yet it was noble and manly when he said: "I do not wish my diploma until, in every particular, I am worthy of it." He was not satisfied to receive a diploma, although he stood high as an operator, and be deficient in other studies, although those studies made him not any more proficient as a filler of teeth, but as a scholar were of importance. Years after merit won, and to-day he enjoys the fruits of his labors, and is an honor to his alumni.

How can the attendance of our association be increased? This question is often asked. In my opinion the small attendance is not caused entirely from outside causes. Dentists, not members, desire its privileges and enjoyments, but through the fear of becoming a member, certain restrictions will conflict with their preconceived ideas of conducting their practice. In one of these States represented here to-day are five hundred dentists and the entire membership of its State Association is only about eighty, and an attendance of fifty members would be considered large. The four hundred and twenty dentists, not members, are largely composed of men from the same schools as the association members, and during college life stood side by side, passed the same professors, and have good standing in society.

What is needed is a great out-reach to those outside of our association, and it may be that some of those minor regulations of its management should be changed; let it be done. Dental colleges graduate students as worthy, why do not the associations receive them? The question has come to this: that the colleges are graduating men whose manner of practice is not in accordance with dental ethics, or the associations are at fault. When the time comes that this important question can be adjusted, and that talent and ability can be commingled, and each to himself derive a personal benefit therefrom, then will good-will and a citizen worthy of respect from his fellow practitioners and the admiration of those in whose midst he lives, be the result.

In conclusion, I would thank the Committee on Programme of the State of Indiana for kindness and courtesies. And may the meeting of this, the Clover Leaf Association, be of great enjoyment, and should we meet again may we not add one more leaf for good luck and include our sister State, Illinois, and thus add the fourth leaf, having the four-leaf clover, emblematic of good-will and prosperity.

